# LCZ696 Therapy Reduces Ventricular Tachyarrhythmia Inducibility in a Myocardial Infarction-Induced Heart Failure Rat Model

**DOI:** 10.1155/2019/6032631

**Published:** 2019-07-01

**Authors:** Po-Cheng Chang, Shien-Fong Lin, Yen Chu, Hung-Ta Wo, Hui-Ling Lee, Yu-Chang Huang, Ming-Shien Wen, Chung-Chuan Chou

**Affiliations:** ^1^Division of Cardiology, Department of Internal Medicine, Chang Gung Memorial Hospital, Linkou, Taiwan; ^2^Chang Gung University College of Medicine, Taiwan; ^3^Institute of Biomedical Engineering, National Chiao Tung University, Hsinchu, Taiwan; ^4^Division of Thoracic Surgery, Chang Gung Memorial Hospital, Linkou, Taiwan; ^5^Department of Anesthesia, Chang Gung Memorial Hospital, Taipei, Taiwan

## Abstract

**Background:**

LCZ696 (valsartan/sacubitril) therapy significantly reduced mortality in patients with heart failure (HF). Although a clinical trial (PARADISE-MI Trial) has been ongoing to examine the effects of LCZ696 in myocardial infarction (MI) patients, the effects of LCZ696 on remodeling of cardiac electrophysiology in animal models remain largely unclear.

**Methods:**

We performed coronary artery ligation to create MI in Sprague-Dawley rats. Echocardiography was performed one week after MI to confirm the development of HF with left ventricular ejection fraction ≤ 40%. MI rats were randomly assigned to receive medical therapy for 4 weeks: LCZ696, enalapril, or vehicle. The sham-operation rats received sham operation without MI creation. In vivo electrophysiological exams were performed under general anesthesia. Western blot analyses were conducted to quantify ion channel proteins.

**Results:**

The HF-vehicle group did not show significant changes in LVEF. Both enalapril and LCZ696 therapy significantly improved LVEF. The HF-vehicle group had higher ventricular arrhythmia (VA) inducibility than the sham group. As compared with the HF-vehicle group, LCZ696 therapy significantly reduced VA inducibility, but enalapril therapy did not. Western blot analyses showed significant downregulation of Na_V_1.5, ERG, KCNE1, and KCNE2 channel proteins in the HF vehicle group compared with the sham group. LCZ696 therapy upregulated protein expression of ERG, KCNE1, and KCNE2.

**Conclusion:**

As compared with enalapril therapy, LCZ696 therapy led to improvement of LVEF, reduced VA inducibility, and upregulated expression of K^+^ channel proteins.

## 1. Introduction

Heart failure (HF) is one of the most frequent diagnoses in patients at admission, with a prevalence of 5.8 million in the United States and over 23 million worldwide [1]. Ventricular tachyarrhythmia is one of the major causes of death in patients with HF [2]. Systolic HF may occur in patients with pressure overload, with volume overload, or following cardiac injury, such as myocardial infarction (MI), hypertension, myocarditis, or drug-induced cardiomyopathy. Among the causes of HF, MI is the top cause of systolic HF in developing and developed countries. Angiotensin-converting-enzyme inhibitors, angiotensin II receptor blockers, beta blockers, and aldosterone antagonists have been widely used in HF patients to improve survival. Even if there had been the remarkable advances of medical therapy in the past decades, HF still carries substantial morbidity and mortality, with a 5-year mortality that is higher than those of many cancers. Ventricular arrhythmias (VAs) and worsening HF account for the major causes of sudden cardiac death in patients with HF. Angiotensin receptor-neprilysin inhibitors are one of the emerging HF pharmacological therapies. In the PARADIGM-HF trial, as compared with enalapril, LCZ696 (valsartan/sacubitril) therapy significantly reduced cardiovascular death and hospitalization for worsening HF in patients with systolic HF [3]. In the LCZ696 therapy group, the reduction of sudden cardiac death contributed to a half of the improvement of survival as compared with the enalapril therapy group, and the reduction of death due to worsening HF contributed to another fourth of the improvement of survival [4]. Although the clinical beneficial effects of LCZ696 are prominent in the PARADIGM-HF trial, whether LCZ696 therapy leads to ion channels remodeling to improve heart function and reduce VAs in infarct-induced HF is largely unknown. Besides the benefits of LCZ696 in patients with systolic HF, the effects of LCZ696 in patients with MI are of more interest to clinicians. Another trial, the PARADISE-MI trial, has been ongoing to examine the effects of LCZ696 in patients with acute myocardial infarction (MI) and HF [5]. Results from the PARADISE-MI study are being expected by all cardiologists. To examine the electrophysiological effects of LCZ696 on post-MI HF, we utilized a MI-induced HF rat model to test our hypotheses: (1) LCZ696 therapy improves left ventricular (LV) systolic function, (2) LCZ696 therapy improves VA inducibility, and (3) LCZ696 therapy leads to ion-channel remodeling.

## 2. Methods

### 2.1. Heart Failure Model Creation and LCZ696 vs. Enalapril Therapy

The research protocol was approved by the Institutional Animal Care and Use Committee (IACUC) of Chang Gung Memorial Hospital and conformed to the Guide for Use of Laboratory Animals (IACUC approval number: 2015011301). Sprague-Dawley rats (BioLASCO Co., Taipei, Taiwan) with a body weight of 250–350 g and an age of 120-210 days were anesthetized with Zoletil (40 mg/kg intraperitoneal), followed by endotracheal intubation with isoflurane (1-1.5%) gas anesthesia. Coronary ligation protocol was conducted to create MI as previously described [6, 7]. The LV was exposed through a left thoracotomy at the fourth or fifth intercostal space. A 6-0 prolene suture was used to ligate the obtuse marginal branches to create MI. The development of MI was documented by one of the presentations of acute MI: ST elevation on the surface electrocardiography (ECG), cyanotic change and hypokinesis of the myocardium of the infarcted myocardium, or scar formation after sacrifice. Control (sham-operation) rats received sham operation without coronary ligation.

**Figure 1:**
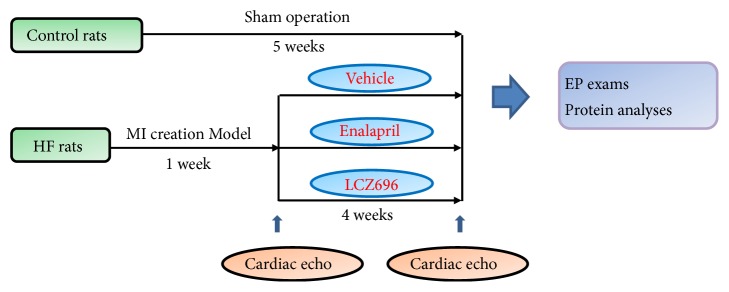
Medical therapy protocol of heart failure (HF)-MI rats. Rats that survived one week after MI created were randomly assigned to receive vehicle, enalapril, or LCZ696 therapy. After 4-week medical therapy, HF-MI rats received electrophysiological (EP) exams to test ventricular arrhythmia (VA) inducibility and then were sacrificed for protein analyses. Sham rats were subjected to EP exams and protein analyses without medical therapy.

After 7-day recovery period following the MI creation, we started the oral medication protocol.  Figure 1 shows the protocol of pharmacological therapy. For the HF-LCZ696 group, LCZ696 (Entresto, Novartis International AG, Basel, Switzerland) was given at a dose of 68 mg/kg/day as described previously [8]. For the HF-enalapril group, enalapril (Renitec, Merck Sharp & Dohme, Kenilworth, NJ, USA) was given at a dose of 20 mg/kg/day [9]. The medications were feed using an awake oral gavage method as previously described [10]. Briefly, medication powder was grounded from oral medication tablets. The certain amount of powder was dispersed in 2 mL of water, and the medication-water mixture was administered using a 10-cm steel gavage feeding needle. In the vehicle group, 2 mL of water was feed using the same oral gavage needle. The duration of medication administration was 4 weeks. Baseline echocardiography was performed 1 week after MI to verify the development of left ventricular systolic dysfunction, which was defined as left ventricular ejection fraction (LVEF) ≤ 40% by using the Teichholz method. Before the echocardiography exams, Zoletil (40 mg/kg intraperitoneal) was administered to anesthetize the rats. A GE Vivid 7 ultrasound machine (GE Healthcare, Chicago, IL, USA) with a pediatric 2D echocardiography GE 10S transducer was used to confirm LV function. A second echocardiography was performed after 4-week medical therapy to evaluate the drugs' effects on left ventricular anatomic and functional remodeling in this model.

### 2.2. In Vivo Electrophysiological Studies

All rats received in vivo electrophysiological studies under general anesthesia using the same protocol during MI creation. Single-lead continuous ECG was recorded using three electrodes placed at the left upper limb, the right upper limb, and the right lower limb. The ECG signals were transmitted to an AxoScope recording device (Axon Digidata 1320A, molecular devices, San Jose, CA, US). A hand-made bipolar electric pacing electrode was placed on the right ventricular free wall epicardium for ventricular stimulation as previously described [11]. The stimuli were delivered with a cardiac electrophysiology stimulator (Bloom DTU 215A, Fischer Medical, Pittsburgh, PA, USA). The output was set at the level of twice threshold. Both burst and extrastimulus pacing protocols were used to test VA inducibility. A burst-pacing protocol was performed at cycle lengths of 200 ms and then down to the shortest 1:1 captured cycle length by 10 ms each step; an extrastimulus pacing protocol was performed at a fixed S1-S1 pacing cycle length of 300 or 250 ms, followed by extrastimuli (up to S5) from 120 ms down to the ventricular effective refractory period (VERP). VA was defined as sustained ventricular rhythm longer than 1 second. Ventricular fibrillation was defined as continuous VA with rapid grossly irregular rhythm, in which particular isolated ventricular beats could not be clearly identified.

### 2.3. Western Blot for Protein Quantification

After electrophysiological studies, the hearts were harvested for protein quantification as previously described [12]. Noninfarcted myocardial tissues from the left ventricles were homogenized and suspended in RIPA buffer containing 20 mM Tris-base (pH 8.0), 150 mM NaCl, 1.0% Nonidet P-40, protease inhibitors (Roche, Basel, Switzerland), and phosphatase inhibitors (Roche, Basel, Switzerland). The total protein concentration in homogenates was determined using a Bradford Assay (Bio-Rad, Hercules, CA, US). After protein concentration determination, the protein samples were subjected to 4-12% SDS-PAGE gel electrophoresis. The proteins in the gel were then electrophoretically transferred to PVDF membranes. The membranes were then incubated in Tween-TBS with the primary antibodies, including a rabbit anti-Na_V_1.5 (AVIVA Systems Biology, San Diego, CA, US), a rabbit anti-  Ca_V_3.1 antibody (Abcam, Cambridge, UK), a rabbit anti-  Ca_V_1.2 antibody (Alomone labs, Jerusalem, Israel), a rabbit anti-K_V_7.1 antibody (Alomone labs, Jerusalem, Israel), a mouse anti-K_V_4.3 antibody (Abcam, Cambridge, UK), a rabbit anti-ERG antibody (Abcam, Cambridge, UK), a rabbit anti-KCNE1 antibody (Proteintech, Chicago, IL, US), a rabbit anti-KCNE2 antibody (Alomone labs, Jerusalem, Israel), a rabbit anti-CX43 antibody (Proteintech, Chicago, IL, US), and a rabbit anti-tubulin antibody (Sigma, St. Louis, MO, US). After the incubation with the primary antibodies, the membrane was then incubated with a horseradish peroxidase-conjugated secondary antibody (Thermo, Waltham, MA, US). The enhanced chemiluminescence films were quantitated by densitometric scanning. The protein expressions were normalized to the expression of tubulin.

### 2.4. Data Analysis

Continuous variables with normal distribution were expressed as the mean ± standard deviation, and categorical variables were expressed as number (percentage). Differences in continuous variables between before-therapy and after-therapy results of the same heart were analyzed by paired Student's t-test. One-way ANOVA with post-hoc LSD analysis was used to compare continuous variables among different groups. Categorical variables were compared using Fisher's exact test. Statistical analyses were performed using IBM SPSS V22.0 (Armonk, NY, USA). The differences were considered significant when the probability value was < 0.05.

## 3. Results

Totally 45 rats (25 males and 20 females) were used in this study: 6 rats received sham operation and 39 rats received coronary artery ligation. Ten of the 39 rats with MI died during the surgery (N=8) or during the first week following development of acute MI (N=2). The remaining post-MI rats received medication therapy according to the aforementioned protocol: 10, 10, and 9 rats received vehicle, LCZ696, and enalapril therapy, respectively. Among these MI rats, 7 (4 males and 3 females, with a mean age of 177 ± 20 days), 9 (5 males and 4 females, with a mean age of 157 ± 23 days), and 6 rats (3 males and 3 females, with a mean age of 161 ± 24 days) survived vehicle, LCZ696, and enalapril therapies. Electrophysiological exams were performed. Six sham rats (3 males and 3 females, with a mean age of 175 ± 21 days) did not receive medication therapy and received electrophysiological exams 5 weeks after surgery. There were no statistical differences in age or gender among the four groups.

### 3.1. LCZ696 and Enalapril Improved Left Ventricular Systolic Function in MI-HF Rats

**Figure 2:**
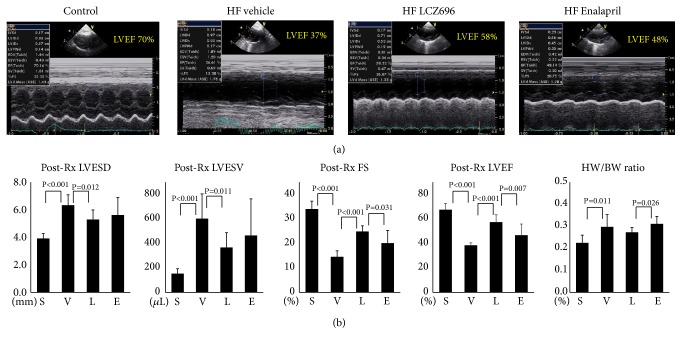
Results of echocardiography exams. (a) Representative examples of echocardiography exams. (b) Summarized results of left ventricular end-systolic diameter (LVESD) and left ventricular end-systolic volume (LVESV), fractional shortening (FS), left ventricular ejection fraction (LVEF), and heart weight (HW)/body weight (BW) ratio. Groups abbreviations: S, sham; V, vehicle; L, LCZ696; E enalapril.

Table 1 shows the summarized results of the cardiac systolic function, chamber sizes, body weights, heart weight ratio, and electrophysiological parameters before and after medical therapy. The sham group had a mean LVEF of 69.1 ± 4.0% (N=6). HF rats that received vehicle therapy (the HF-vehicle group) had no significant improvement in LVEF (36.2±6.9% at baseline to 38.5±2.0% after therapy, N=7, P=0.188). Both HF-enalapril and HF-LCZ696 therapies significantly improved LVEF (the HF-enalapril group: 37.7±2.8% to 46.7±9.1%, N=6, P=0.030; the HF LCZ696 group: 36.9±4.5% to 57.6±5.5%, N=9, P<0.001) and fractional shortening (the HF-enalapril group: 7.4±0.9% to 20.1±5.2%, N=6, P=0.003; the HF-LCZ696 group: 7.6±1.2% to 26.2±3.8%, P<0.001). The HF vehicle group had significantly a heavier mean heart weight than the sham group (1.66±0.25g, N=7 vs. 1.29±0.18g, N=6, P=0.008). There was a trend toward lighter heart weight in the HF-LCZ696 therapy group (1.45±0.11g, N=9, P=0.058) than that in the HF-vehicle group. As compared with the HF-vehicle group, the HF-LCZ696 group has lower LV end-systolic diameter (LVESD, 5.3±0.7 mm vs. 6.3±0.8 mm, P=0.012) and LV end-systolic volume (LVESV, 367±121 *μ*l vs. 602±202 *μ*l, P=0.011). The HF-vehicle group (0.31±0.05%) had a significantly higher heart weight/body weight (HW/BW) ratio than the sham group (0.22±0.04%, P= 0.003). The HF-LCZ696 group (0.27±0.02%) had a lower HW/BW ratio than the HF-enalapril group (0.31±0.03%, P=0.026).  Figure 2(a) shows representative examples of echocardiographic exams, and Figure 2(b) shows the summarized results of posttherapy heart chamber sizes, fractional shortening, LVEF, and HW/BW ratio.

### 3.2. LCZ696 Ameliorated Inducible Ventricular Tachyarrhythmias in MI-HF Rats

Figure 2(b) shows the bar graphs of the electrophysiological exams and Table 1 summarized the mean values of the results. The HF-vehicle group had a longer corrected QT interval (260±35 ms, N=7 vs. 227±13 ms, N=6, P=0.036) and a longer ventricular effective refractory period (VERP) (65.7±9.8 ms vs. 56.7±5.2mg, P=0.047) than the sham group. LCZ696 therapy attenuated the prolongation of VERP (53.8±5.2ms, N=9, P=0.010), but enalapril therapy did not alter the VERP (60.0±6.3ms, N=6, P=0.246). As compared with the enalapril group, there was a trend toward a shorter VERP in the LCZ696 group (P = 0.064).

**Figure 3:**
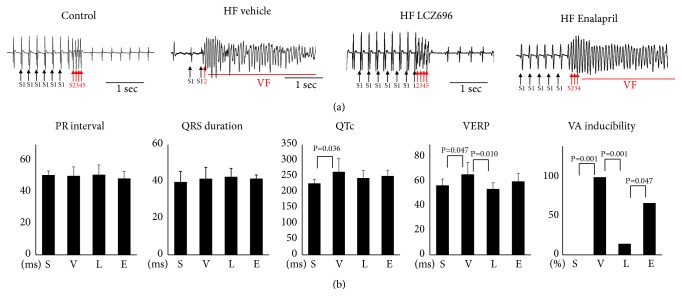
Results of electrophysiology exams. (a) Representative examples of echocardiography exams. (b) Summarized results of PR interval, QRS duration, corrected QT interval (QTc), ventricular effective refractory period (VERP), and ventricular arrhythmia (VA) inducibility. Groups abbreviations: S, Sham; V, vehicle; L, LCZ696; E enalapril.

The electrophysiological studies showed that VA was not inducible in the sham group and was inducible in all rats in the HF vehicle group (0%, N=6 vs. 100%, N=7, P=0.001). Enalapril therapy did not significantly reduce the VA inducibility (67%, N=6 vs. 100%, P=0.227), but LCZ696 therapy significantly reduced the VA inducibility (11%, N=9, vs. 100%, P=0.001). In addition, the HF-LCZ696 group showed a lower VA inducibility than the HF enalapril group (11% vs. 67%, P=0.047).  Figure 3(a) shows the representative ECG tracings of VA inducibility tests, and Figure 3(b) shows the bar graph or the summarized results of the electrophysiological studies.

### 3.3. LCZ696 Reversed Downregulation of Potassium Channels in MI-HF Rats

**Figure 4:**
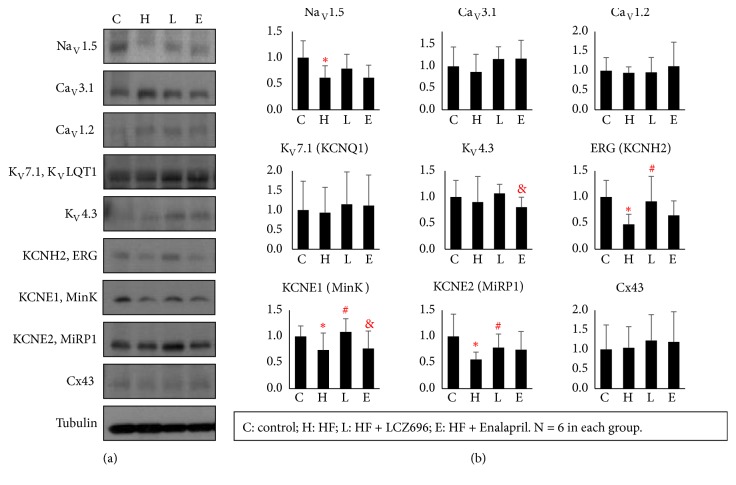
Results of protein analyses. (a) Representative examples of channel proteins. (b) Summarized results of channel proteins. Groups abbreviations: S, Sham; V, vehicle; L, LCZ696; E enalapril. N = 6 for all groups. *∗* indicates P < 0.05 in the comparison between the sham group and the HF-vehicle group. # indicates P < 0.05 in the comparison between the HF-vehicle group and the HF-LCZ696 group. & indicates P < 0.05 in the comparison between the HF-vehicle group and the HF-LCZ696 group.

Ion channel remodeling plays an important role in the electrophysiological changes. We further performed Western blot to quantify ion channel protein expression (n = 6 for each group). The results are shown in Figure 4 and Table 2. The HF group expressed significant lower concentrations of Na_V_1.5 (0.62±0.23AU vs. 1.00±0.32AU, P=0.020), KCNE1 (0.74±0.24AU vs. 1.00±0.20AU, P=0.018), KCNE2 (0.62±0.14AU vs. 1.00±0.43AU, P=0.020), and ERG (0.48±0.19AU vs. 1.00±0.32AU, P=0.003) than the sham group. LCZ696 therapy significantly increased the protein expression of KCNE1 (1.08±0.25AU vs. 0.74±0.24AU, P=0.018), KCNE2 (0.79±0.26AU vs. 0.56±0.14AU, P=0.044), and ERG (0.92±0.48AU vs. 0.48±0.19AU, P=0.030). There was a trend of increased Na_V_1.5 expression (0.79±0.27AU vs. 0.62±0.23AU, P=0.130) after LCZ696 therapy, but the comparison was not statistically significant. The protein expression of Cx43, Ca_V_1.2, and Ca_V_3.1 was not different among these 4 groups.

## 4. Discussions

The effects of LCZ696 therapy in this rat MI-induced HF model include reverse remodeling of cardiac hypertrophy, improvement of LVEF, and reduced arrhythmia inducibility in the in vivo electrophysiological study. As compared with the HF-vehicle group, enalapril therapy led to improvement of LVEF. LCZ696 therapy further improved LVEF and reduced VA inducibility as compared with enalapril therapy. Protein analyses showed significant downregulation of Na_V_1.5, ERG, KCNE1, and KCNE2 expressions in the HF-vehicle group as compared with the sham group. LCZ696 therapy also led to reverse remodeling of several K^+^ channels, which were upregulated expression of ERG, KCNE1, and KCNE2 in this MI-HF rat model.

### 4.1. The Effects of LCZ696 Therapy on Ventricular Function and Cardiac Remodeling

In this study, LCZ696 therapy caused improvement of LVEF and reduction of heart weight. LVEF is significantly associated with prognoses in patients with systolic heart failure. Clinical therapies which help LVEF improvement usually lead to a better prognosis. A meta-analyses report showed a trend of association between improvement of LVEF and better survival in patients who received percutaneous coronary intervention after acute MI [13]. Since MI is the top cause of systolic HF in developing and developed countries, clinical therapies in the HF patients with MI are of interest to cardiologists. In a previous study, therapy with angiotensin converting enzyme inhibitors in patients with acute MI showed benefits on survival [14]. Although the PARADIGM-HF trial demonstrated a further improvement cardiovascular mortality and HF hospitalization in the LCZ696 treatment group as compared with the enalapril group [3], the clinical outcomes in post-MI HF patients who received LCZ696 therapy remain unclear. To answer the question, another prospective randomized clinical trial (PARADISE-MI) to compare the outcomes of LCZ696 therapy and ramipril therapy in patients with HF events after MI has been ongoing.

We conducted this animal model study and hopefully the results may reflect the outcome prospects of the PARADISE-MI trial. In this study, we demonstrated that both enalapril and LCZ696 improved LVEF and that LCZ696 therapy led to a greater effect on LVEF. As compared with enalapril therapy, the additional effects of LCZ696 may be attributed to inhibition of cardiac fibrosis [15], suppression of proinflammatory cytokine [16], and enhancement of nitric oxide (NO) bioavailability [17]. In a report by von Lueder et al., LCZ696 therapy reduced degree of fibrosis in noninfarct remote myocardium in the peri-infarct zone [15]. Although LCZ696 did not reduce the infarct size, attenuated degree of fibrosis in the viable myocardium may be one of the mechanisms of LVEF improvement after LCZ696 therapy. The effects of LCZ696 on cardiac remodeling in this study are also compatible with the clinical benefits of LCZ696 in patients with HF [3].

### 4.2. The Effects of LCZ696 Therapy on Cardiac Repolarization Reserve

Besides worsening HF due to left ventricular dysfunction, ventricular tachyarrhythmia is one of the most important causes of mortality in patients with HF. In the PARADIGM-HF trial, as compared to the traditional enalapril therapy, the LCZ696 therapy led to an absolute reduction of cardiovascular death rate by 3.2% (from 16.5% to 13.3%) [4]. A study by de Diego C et al. also revealed that LCZ696 therapy significantly decreased ventricular tachycardia and reduced appropriate implantable cardioverter-defibrillator shocks [18]. This study revealed that LCZ696 therapy reduced VA inducibility in the rat MI-HF model, indicating that the electrophysiological remodeling might be the major cause of reduced cardiovascular death in the PARADIGM-HF trial and the study performed by de Diego C et al. The mechanisms of the reduced VA inducibility include recovery of systolic function, reduced fibrosis, reduced ventricular wall stress, and ion channel remodeling. Patients with HF have prolonged QT interval and impaired repolarization reserve [19]. This study showed that QTc interval was significantly longer in the HF rats than the sham rats. There was a trend toward shorter QTc interval after LCZ696 therapy. In addition, the ventricular ERP of the LCZ696-treated HF rats was significantly shorter than that of the vehicle-treated HF rats, suggesting a shorter action potential duration in the HF-LCZ696 group. The electrophysiological remodeling following LCZ696 therapy thus partly explained the mechanisms of reduced arrhythmias in human studies. The long-term (4-week) effects of LCZ696 treatment may differ from the acute effects of LCZ696. It has been reported that single dose of LCZ696 did not affect cardiac repolarization in healthy male subjects [20]. The reverse of electrophysiological remodeling after 4-week LCZ696 therapy may reflect the improvement of LVEF and remodeling of ion channel proteins rather than acute effects on transmembrane ion channel currents.

### 4.3. The Effects of LCZ696 Therapy on Remodeling of Ion Channel Proteins

**Figure 5:**
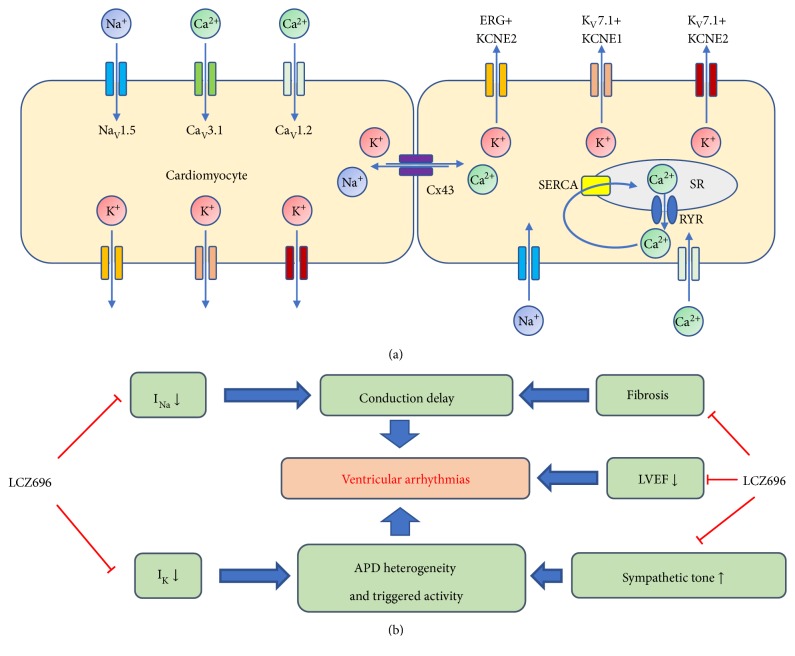
(a) Action potential generation associated ion channel proteins test in this study. (b) Summarized diagram of the effects of LCZ696 therapy on the HF-MI rat model. APD, action potential duration; I_K_, potassium current; I_Na_, sodium current; SERCA, sarco/endoplasmic reticulum Ca^2+^-ATPase; SR, sarcoplasmic reticulum; RYR, ryanodine receptor.

In this study, LCZ696 therapy increased the expressions of potassium channels in this rat MI-HF model, including ERG, KCNE1, and KCNE2. These ion channels are responsible for rapidly rectifying delayed potassium current (I_Kr_) and slowly rectifying delayed potassium current (I_Ks_) in cardiomyocytes [21], which are associated with long QT syndromes (types 2, 5, and 6). These major myocardial potassium currents (I_Kr_, I_Ks_, and I_To_) are involved in the repolarization of myocardial action potential [22]. Reduction of those ion channel functions is associated with ventricular tachyarrhythmias and Torsades de Pointes. Downregulation of the major potassium channels has been observed in HF and MI [23]. The alterations in potassium channels and NCX may decrease repolarization reserve and increase inward currents, leading to arrhythmogenesis in failing hearts. In this study, the reverse remodeling of ERG, KCNE1, and KCNE2 with LCZ696 therapy may contribute, at least in part, to amelioration of VA inducibility. The upregulation of potassium channels led to reverse of prolonged QT interval and VERP in the MI-HF myocardium and arrhythmogenesis in the diseased hearts. The roles of these ion channel proteins on generation of the cardiomyocyte action potential are shown in Figure 5(a). LCZ696 therapy led to attenuation of downregulation of the major potassium channel protein, subsequently leading to recovery of prolonged VERP. In conjunction with amelioration of cardiac HF remodeling and improvement of LVEF, LCZ696 therapy reduced ventricular arrhythmia inducibility (Figure 5(b)).

### 4.4. Mechanisms of LCZ696 Therapy to Reduce Cardiac Death

In the PARADIGM-HF trial, LCZ696 therapy reduced the risk of cardiac death by 19% (absolute risk reduction 3.2%, from 16.5% to 13.3%) and the risk of hospitalization for heart failure by 21% (absolute risk reduction 2.8%, from 15.6% to 12.8%) [3]. The reduction of hospitalization for worsening HF can be explained by the recovery of LVEF, suppression of proinflammatory factors, and reduction of fibrosis with LCZ696 therapy. The improvement of LVEF, the recovery of electrophysiological remodeling, ion channel proteins and myocardial fibrosis [16] may explain the reduced VA inducibility, which may explain the mechanisms of decreased sudden cardiac death in the PARADIGM-HF Trial.

### 4.5. Mechanisms of Neprilysin Inhibition on Cardiac Systolic Function and Electrophysiology

The mechanisms of LCZ696-associated reverse cardiac remodeling and amelioration of electrophysiological changes had not been well understood yet. As compared with enalapril, LCZ696 has additional neprilysin inhibitory effects. Neprilysin is an endogenous endopeptidase, which breaks down natriuretic peptides, bradykinin, substance P, angiotensin II, and a number of other peptides. Neprilysin inhibition leads to diuresis, vasodilatation, reduced sympathetic nerve activity, inhibition of cardiac hypertrophy, suppression of apoptosis, and inhibition of fibrosis [24]. Because neprilysin inhibition involves variable peptides and many pathways, the precise mechanisms of the effects of neprilysin inhibition are very complex and are not well defined. For example, increased circulating natriuretics peptides lead to increased cyclic GMP, which in turn alters protein expression of ion channels. GMP-regulated transcription factors include the cAMP-response element binding protein (CREB), the serum response factor (SRF), and the nuclear factor of activated T cells (NF/AT) [25]. In addition to the inhibitory effects on natriuretic peptides, LCZ696 also has the effects of sympathetic nerve activity inhibition, which in turn influences protein expression through activation of its mineralocorticoid receptor (MR) and G protein-coupled receptor (GPCR) signaling [26]. Recently, Iborra-Egea O et al. used Therapeutic Performance Mapping System technology to analyze the possible mechanisms involved in the synergistic effects of valsartan and sacubitril [27]. Most of the potential synergistic nodes, such as AKT1, AKT3, FAK1, CSK3B, PK3CA, FGF2, MMP3, and TGFB1, are associated with cardiomyocyte cell death and ventricular extracellular matrix remodeling. Although the mapping study depicted a potential of the mechanisms of LCZ696 therapeutic effects, further studies are necessary in order to define the causal associations.

## 5. Limitations

In the enalapril and LCZ696 groups, rats received high dosages of pharmacologic therapy (LCZ696 at a dose of 68 mg/kg/day and enalapril at a dose of 20 mg/kg/day). The dosages could not be comparable with the clinical dosages in human. Although the HF LCZ group had a trend toward reduced sudden death during the 4-week therapy period, the design of this study was not to test whether LCZ696 therapy improved survival and thus the statistical analyses did not reach significant difference.

## 6. Conclusions

In this MI-HF rat model, as compared with enalapril therapy, LCZ696 therapy led to a greater improvement of LVEF and lower VA inducibility. Protein analyses showed significant downregulation of Na_V_1.5, ERG, KCNE1, and KCNE2 expression in the HF vehicle therapy rats. LCZ696 therapy increased ERG, KCNE1, and KCNE2 expression. Enalapril therapy also led to improvement of LVEF but did not significantly improve the VA inducibility and did not alter the expression of potassium ion channels. The significant improvement of systolic function, electrophysiological benefits, and reverse remodeling of protein expression in the HF-LCZ696 group may explain the reduction of VA inducibility.

## Figures and Tables

**Table 1:** Echocardiographic, electrophysiological, and anatomic parameters.

Parameters	Sham	HF vehicle	HF LCZ696	HF enalapril	P value	P value	P value	P value
	(N = 6)	(N = 7)	(N = 9)	(N = 6)	V vs. S	L vs. V	E vs. V	L vs. E
Baseline LVEF (%)	NA	36.2 ± 6.9	36.9 ± 4.5	37.7 ± 2.8	NA	0.797	0.618	0.684
Post-Rx LVESD (mm)	3.9 ± 0.4	6.3 ± 0.8	5.3 ± 0.7	5.6 ± 1.3	<0.001	0.012	0.239	0.530
Post-Rx LVESV (*μ*l)	153 ± 40	602 ± 202	367 ± 121	465 ± 299	<0.001	0.011	0.345	0.389
Post-Rx FS (%)	34.0 ± 3.2	14.5 ± 2.5	24.8 ± 2.3	20.1 ± 5.2	<0.001	<0.001	0.027	0.031
Post-Rx LVEF (%)	69.1 ± 4.0	38.5 ± 2.0	57.6 ± 5.5	46.7 ± 9.1	< 0.001	< 0.001	0.040	0.007
Body weight (g)	578 ± 43	562 ± 55	527 ± 20	500 ± 49	0.562	0.095	0.057	0.163
HW/BW ratio (%)	0.22 ± 0.04	0.30 ± 0.05	0.27 ± 0.02	0.30 ± 0.03	0.003	0.041	0.886	0.016
Heart weight (g)	1.29 ± 0.18	1.66 ± 0.25	1.45 ± 0.11	1.55 ± 0.18	0.008	0.058	0.396	0.237
Heart rate (BPM)	213 ± 34	210 ± 51	214 ± 28	224 ± 24	0.888	0.848	0.552	0.502
PR interval (ms)	50.7 ± 2.6	50.1 ± 5.7	50.9 ± 6.2	48.5 ± 4.4	0.825	0.817	0.577	0.444
QRS duration (ms)	39.7 ± 5.6	41.4 ± 6.2	42.5 ± 4.6	41.5 ± 2.0	0.575	0.709	0.979	0.631
Corrected QT (ms)	227 ± 13	260 ± 35	244 ± 25	253 ± 23	0.036	0.305	0.695	0.459
VERP (ms)	56.7 ± 5.2	65.7 ± 9.8	53.8 ± 5.2	60.0 ± 6.3	0.047	0.010	0.246	0.064
VA inducibility (%)	0 (0%)	7 (100%)	1 (11%)	4 (67%)	0.001	0.001	0.227	0.047

BPM, beats per minute; FS, fractional shortening; HW/BW, heart weight/body weight; HF, heart failure; NA, not available; Post-Rx, postmedical therapy (or before electrophysiological exam in the sham group); VA, ventricular tachyarrhythmia; VERP, ventricular effective refractory period.

Groups abbreviations: S, Sham; V, vehicle; L, LCZ696; E, enalapril.

**Table 2:** Protein expression.

Protein expression	Sham	HF	HF	HF	P value	P value	P value	P value
		vehicle	LCZ696	enalapril	V vs. S	L vs. V	E vs. V	L cs E
Na_V_1.5	1.00 ± 0.32	0.62 ± 0.23	0.79 ± 0.27	0.62 ± 0.24	0.020	0.130	0.497	0.136
Ca_V_3.1	1.00 ± 0.45	0.87 ± 0.41	1.17 ± 0.28	1.18 ± 0.42	0.307	0.085	0.114	0.485
Ca_V_1.2	1.00 ± 0.34	0.94 ± 0.16	0.96 ± 0.38	1.11 ± 0.62	0.402	0.475	0.333	0.365
K_V_7.1 (KCNQ1)	1.00 ± 0.74	0.93 ± 0.65	1.15 ± 0.83	1.12 ± 0.78	0.437	0.316	0.335	0.474
K_V_4.3	1.00 ± 0.32	0.91 ± 0.49	1.07 ± 0.17	0.81 ± 0.19	0.349	0.227	0.326	0.016
ERG (KCNH2)	1.00 ± 0.32	0.48 ± 0.19	0.92 ± 0.48	0.65 ± 0.28	0.003	0.030	0.120	0.129
KCNE1 (MinK)	1.00 ± 0.20	0.74 ± 0.27	1.08 ± 0.25	0.77 ± 0.34	0.043	0.023	0.435	0.048
KCNE2 (MiRP1)	1.00 ± 0.43	0.56 ± 0.14	0.79 ± 0.26	0.74 ± 0.35	0.018	0.044	0.127	0.410
Cx34	1.00 ± 0.63	1.05 ± 0.54	1.23 ± 0.66	1.19 ± 0.77	0.448	0.304	0.354	0.465

Groups abbreviations: S, Sham; V, vehicle; L, LCZ696; E, enalapril. N = 6 for each group.

## Data Availability

Please refer to https://figshare.com/s/99c84f573aa9a889c512.
